# Needle-free connectors catheter-related bloodstream infections: a prospective randomized controlled trial

**DOI:** 10.1186/s40635-019-0277-7

**Published:** 2019-12-02

**Authors:** Michael Koeppen, Franziska Weinert, Sabrina Oehlschlaeger, Andreas Koerner, Peter Rosenberger, Helene Anna Haeberle

**Affiliations:** 1 Department of Anesthesiology and Intensive Care Medicine, Universitätsklinikum Tübingen, Hoppe-Seyler-Straße 3, 72076 Tübingen, Germany; 2 Department of Anesthesiology, Klinikum Sindelfingen-Böblingen, Böblingen, Germany; 3 Department of Cardiac Surgery, University Hospital, Tübingen, Germany

**Keywords:** Needle-free connectors, Catheter-related bloodstream infections, Bionecteur®

## Abstract

**Background:**

In the critically ill, catheter-related bloodstream infection can result from bacterial contamination of infusion hubs of intravascular catheters. Needle-free connectors (NFC) have been suggested to reduce the rate of bacterial contamination and subsequent catheter-related bloodstream infection (CRBSI), but data remains ambiguous. Thus, we tested if a novel NFC would reduce bacterial contamination and subsequent CRBSI.

**Results:**

In a prospective, randomized controlled trial, surgical ICU patients were randomized to three-way hubs closed by caps or Bionecteur® (Vygon, Inc.) of central venous catheters. Every 72 h, infusion lines were renewed and microbiological samples were taken. Bacterial growth was analyzed by blinded microbiologists. Incidence of bacterial contamination and CRSBI were assessed. Outcome parameters like length of stay on ICU and outcome were retrospectively assessed. Two thousand seven hundred patients were screened, 111 were randomized to the NFC, and 109 into the control group. Finally, 24 patients in the NFC and 23 control patients were analyzed. The majority of samples (NFC 77%; control 70%) found no bacterial growth. Coagulase-negative staphylococci were most commonly detected on CVC samples (NFC 17%; control 21%). We found CRBSI (defined as identical pathogens in blood culture and catheter line tip culture, and clinical manifestations of infection) in two control patients and one patient of the NFC group. Their length of ICU stay did not differ between groups (NFC 19 days; control 23 days).

**Conclusion:**

The use of NFC does not influence the rate of bacterial contamination of infusion hubs of central venous catheters.

**Trial registration:**

**Clinicaltrials.gov**, **NCT02134769**. Registered 09 May 2014.

## Highlights

Needle-free connectors are safe in a surgical setting.

## Background

Catheter-related bloodstream infections (CRBSI) are preventable, but common healthcare-associated infections. Apart from increasing the length of intensive care unit (ICU) treatment, they are associated with the risk to cause life-threating infections. Common ports of entry for the infectious agents are indwelling intravascular catheters, such as arterial lines and central venous catheters (CVC). The treatment with peripherally incompatible infusions (e.g., hyperosmolar drugs, sedatives, parental nutrition) requires a CVC placement. CVC-related infections occur on 1.02 days per 1000 CVC-days [1] and 60% of intensive care unit (ICU) patients require a CVC. New strategies to reduce contamination via an indwelling central venous or arterial catheter could, therefore, improve patient outcome.

Before the actual bloodstream infections occur, intravascular catheter entry ports often become contaminated with bacteria. Such contaminations occur during handling of intravascular line connectors during the connection of infusions, injections of drugs or blood sample withdrawal. Especially multivolume catheters, therefore, bear a higher risk of infection, due to the higher frequency of connections and disconnections of infusions [2]. Multiple studies have evaluated measurements to reduce CVC contaminations using sterile dressings, anti-infective coatings, antibiotic-impregnated catheters and multi-step bundles consisting of washing procedures with antiseptic solutions. In Germany, the commission of the federal government for hospital hygiene and infection prevention (*Kommission für Krankenhaushygiene und Infektionsprävention, Robert-Koch-Institut; RKI*) recommends the use of needle-free connectors (NFC) to reduce the risk of infection. This recommendation relies on the rationale, that NFC may reduce manipulation on the CVC connections and therefore reduce the time microorganism can contaminate the ports [3]. If NFC decrease CRBSI remains a matter of debate, since some trials found no impact on infections, whereas case-series found an increase in systemic infections [4, 5].

To test, if NFC usage could influence CRBSI rates in surgical patients, we performed a prospective randomized trial using a novel needleless connector (Bionecteur®, Vygon Inc.) in surgical ICU setting, and analyzed repetitive microbiological samplings of the connectors on arterial lines and central venous catheters as a surrogate for bacterial contamination and blood cultures.

## Methods

### Ethics

This prospective clinical trial was approved by the Ethics Committee of the University Hospital Tuebingen prior to recruitment (394/2014MPG23). Before randomization, written consent was acquired from all patients or their legal representative prior to study inclusion. If the patient was incapable to provide informed consent but no advanced directive regarding the legal representative was made, a formal legal guardianship was requested at the respective dependency court. If a legally binding informed consent was absent prior to the first study intervention, the patients were excluded from the study. The study was registered in Clinical Trial Database (NCT02134769).

### Study setting, population and sample size

The study was performed as a simple, single-center randomized controlled trial at tertiary, university hospital intensive care unit. The study included patients requiring a central venous catheter or an arterial cannula for monitoring or therapy.

### Inclusion and exclusion criteria

Inclusion criteria were 18 years of age or older, admission to the intensive care unit, the necessity for a central venous catheter or arterial line, anticipated ICU treatment for more than 5 days and informed consent by the legal representative. Exclusion criteria included ICU stay for less than 3 days (including non-survival), positive blood cultures prior to first infusion line change, the unknown time point of catheter placement, catheter placement at a referring hospital or absence of informed consent. Patients with intellectual disability were also excluded from the study.

### Primary and secondary endpoint

The primary endpoint of our study was the number of CRBSI. Secondary endpoints were bacterial colonization of intravascular catheter line entry ports.

### Definition of catheter-related bloodstream infections and clinical criteria

In line with international guidelines [6, 7], CRBSI was diagnosed when the following criteria were met: bacteremia/fungemia in a patient with an intravascular catheter with at least one positive blood culture obtained from a peripheral vein, catheter tip colonization, no apparent source for the bloodstream infection except the catheter and clinical manifestations of infection. Clinical criteria for an infection were fever (body temperature > 38 °C), tachycardia, tachypnea, hypotension or increase of vasopressors by more than 50% in 24 h, chills in the presence of elevated inflammatory markers (C-reactive protein > 5 mg/dl postoperatively, procalcitonin > 0.5 ng/ml).

### Study protocol and randomization

**Fig. 1:**
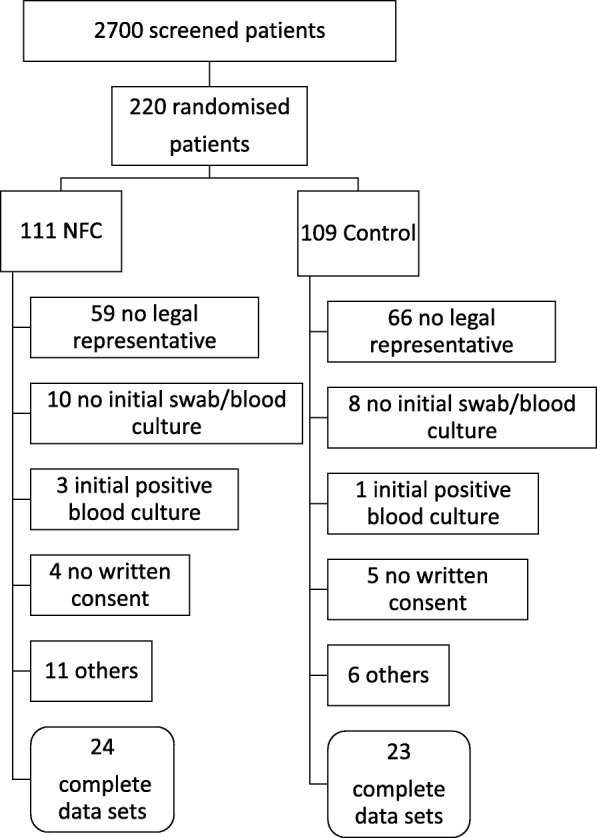
Flow chart of screening and randomization

Intravascular catheters were either placed in the operating room just prior to ICU admission or after ICU admission under sterile conditions. Patients received intravascular catheter solely for the management of the underlying disease. On day 3 after ICU admission, the attending intensivist on call screened patients for inclusion or exclusion criteria and enrolled the patients if applicable. If patients were included in the study, patients were randomized into either the NFC group or the control group. Numbered, concealed envelopes were used for randomization based on a randomization list generated by the Institute of Biometrics at the University of Tuebingen. In the treatment group, we replaced caps by NFCs (Fig. 1). All staff involved in patient care received training in the use of NFCs used in our study (Vygon Bionecteur®) prior to the study.

The study protocol entailed a programmed change of the central venous pressure infusion lines and arterial pressure infusion line every 72 h. We collected a set of blood cultures prior to the change of infusion lines for the first time. Simultaneously to infusion line changes, microbiological samples were collected from each port of the CVC or arterial lines. Before each handling of the ports, spray disinfection before application of medication or before changing infusion lines was performed. All infusion hubs of a four-way stopcock were capped by a sterile cap.

Microbiological analysis was performed by the Institute of Medical Microbiology at the University Tuebingen according to routine standard-operating procedures. The medical microbiologist was blinded to the study group.

### Staff training

Before the study, all nursing staff and physicians of our intensive care unit were trained in the handling of NFCs. In a first step, the manufacturer of Bionecteur, Vygon Inc®, trained senior critical care nurses, who also hold a certificate as practical nursing trainers. Then, the nursing care trainers served as proxies and subsequently trained all other staff within a 4 weeks’ time frame. The study protocol was initiated after all personnel had completed training in NFC handling.

### Access-ports nursing care

Before handling, NFCs were sprayed with a 70% propranolol-based antiseptic solution and then scrubbed for 15 s. After an additional wait period of 15 s to allow the antiseptic solution to dry, the NFC entry ports were then handled.

In the control group, the three-way stopcocks were also sprayed with a 70% propranolol-based antiseptic solution after uncapping and before infusion lines were connected or medication injected. A new, sterilely packed end-cap was placed on the three-way stopcock after each handling.

Regardless of the study group, both systems were flushed with a bolus of several milliliters of normal saline after injection of medication.

### Statistical analysis

Statistical planning and evaluation were performed by the Institute of Biometrics at the University of Tuebingen. The data were analyzed by Mann-Whitney and *T* test. A *p* value of < 0.05 was considered significant.

## Results

### Patients

During the planning phase, a sample size of 300 patients (150 per group) was calculated, based on the incidence of CRSBI in surgical ICUs. Between April 2014 and October 2016, 2700 patients were screened. A total of 2480 patients met the exclusion criteria. Trial recruitment ended at the pre-defined deadline in October 2016. Two hundred twenty patients were randomized. Exclusion criteria were fulfilled in 87 patients in the NFC group and 86 patients in the control group (Fig. 1). The main reason for exclusion was the absence of a legal representative. Ultimately, we analyzed data from 24 patients in the NFC group and 23 patients.

The patient population did not differ in basic demographics (e.g., age or gender) or regarding co-morbidities (Table 1). Most of the patients were admitted for postoperative surveillance and critical care management after cardiac surgery. Pneumonia was common in both groups on admission. Taken together, the NFC and the control group showed comparable baseline characteristics.

**Table 1:** Demographics

	NFC (*n* = 24)	Controls (*n* = 23)	*p* values
Age—year (mean ± SD)	64.2 ± 16.8	65.5 ± 11.3	0.76
Male sex—no. (%)	15 (58)	13 (57)	0.76
Co-morbidities			
Coronary artery disease	7 (30.4)	5 (20.8)	0.06
Arterial hypertension	15 (65.2)	19 (79.2)	0.19
Chronic obstructive pulmonary disease	1 (4.3)	2 (8.3)	0.61
Diabetes mellitus type II	6 (26.1)	8 (33.3)	0.53
Admitting surgical specialty			
Cardiothoracic surgery (%)	11 (41)	16 (59)	0.14
General surgery (%)	6 (25)	5 (22)	> 0.99
Other	6 (25)	2 (9)	0.24
ICU admission diagnosis			
Postoperative surveillance after cardiac surgery (%)	10 (43.5)	9 (37.7)	> 0.99
Abdominal surgery (laparotomy)	4 (17.4)	1 (4.2)	0.35
Intracranial hemorrhage	0	3 (12.5)	0.11
Acute respiratory distress syndrome	2 (8.7)	2 (8.3)	0.99
Sepsis	0	1 (4.2)	0.49
Pneumonia	18 (78.3)	14 (58.3)	0.36
SIRS	3 (13)	2 (8.3)	> 0.99
Peritonitis	3 (13)	1 (4.2)	0.61
Urinary tract infection	3 (13)	4 (16.6)	0.70
Others	7 (30.4)	8 (33.3)	0.76

### Change of infusion lines, arterial or central venous catheters

After having defined the general characteristics, we analyzed the variables increasing the risk for CRBSI. Even though patients in the control group stayed longer in the ICU (median 23 days) than patients in the NFC group (19 days), the difference was not significant. In 34.8% of the control patients and in 29.1% of the NFC group more than one new placement of a CVC line was necessary. On average, CVC remained in situ for an average of 10.6 ± 5.8 days in the NFC or 10.4 ± 4.2 days in the control population (*p* = 0.89) (Table 2).

**Table 2:** ICU variables and microbiological sampling parameters

	NFC (*n* = 24)	Controls (*n* = 23)	*p* values
Median days length of ICU stay (interquartile range)	19 (11.5–29.0)	23 (15.0–31.00)	0.41
Median number of infusion line changes (interquartile range)	5 (3–8.5)	7 (4–8)	0.47
Median number of CVC changes (interquartile range)	2 (1–2.75)	2 (2–3)	0.06
Median days of CVC in situ (interquartile range)	9.5 (6–15)	10 (8–14)	0.89
Median number of arterial line changes (interquartile range)	1 (0–2)	1 (0–2)	0.73
Central venous catheter location			
Jugular vein	41	48	0.81
Subclavian vein	7	12	0.45
Femoral vein	2	0	0.20
Microbiological sampling			
Median number of entry port sampling (interquartile range)	5 (2–7)	4 (3–6)	0.61
Number of blood cultures collected (%)	17 (71)	18 (78)	0.74
Blood cultures tested positive (% of all)	2 (12)	4 (22)	0.65

Our study protocol did not specify the preferred location for the CVC placement., but CVC location influences the rate of infection [8]. Thus, we analyzed if the control or the NFC population differed in the site of central venous catheter placement. Most commonly, CVC was most commonly placed in the jugular vein (Table 2). Only on two occasions, the femoral vein was used for CVC placement in the NFC. No difference was detected between the groups. Thus, NFC usage did not influence the number of CVC placements or catheter locations.

### Microbiological sampling results

**Fig. 2:**
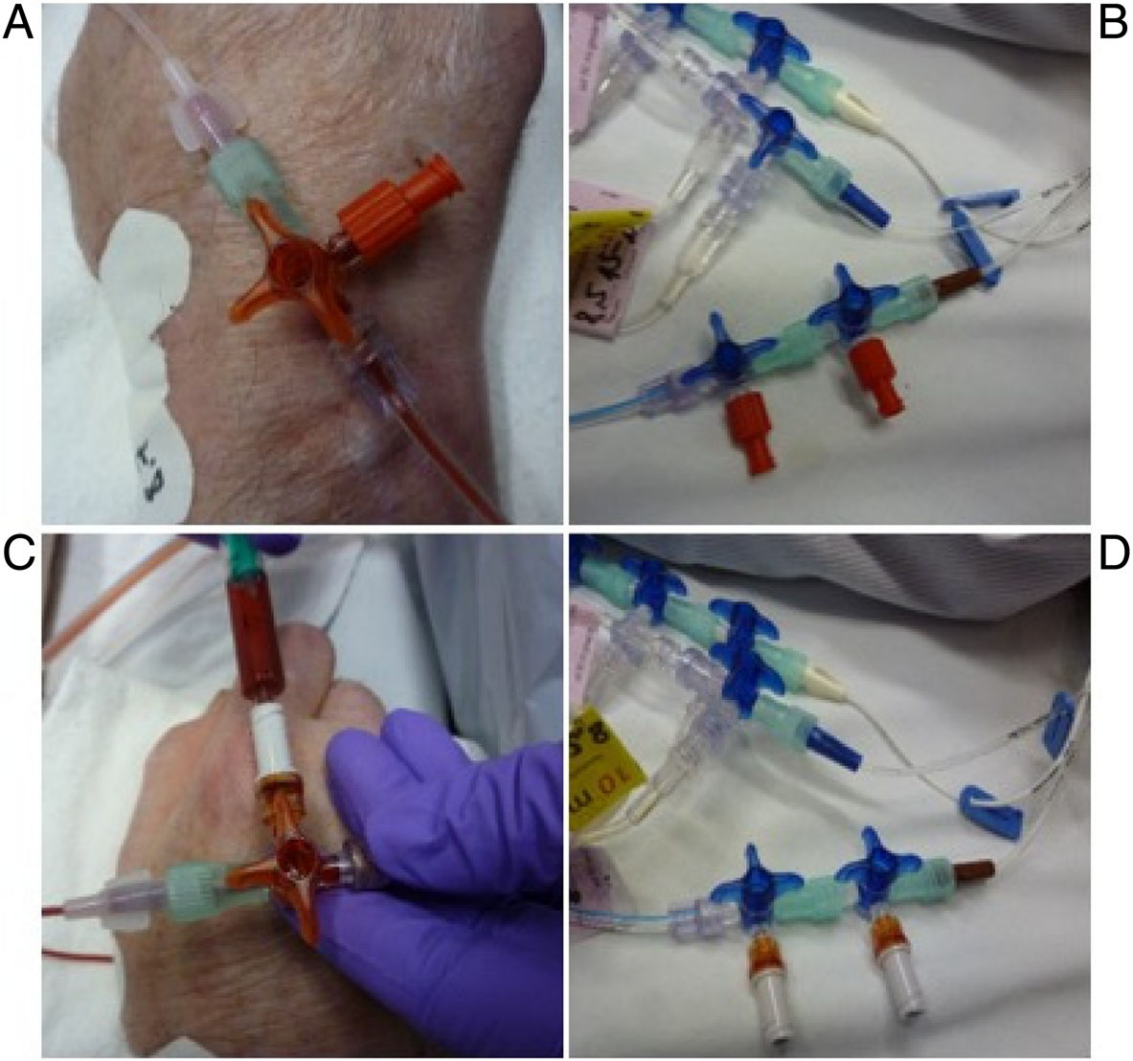
Setup of entry port connectors. **a** Arterial connection before the implementation of NFC; **b** CVC connection before the implementation of NFC including stopcocks and caps; **c** Arterial connection including Bionecteur; **d** CVC connection using Bionecteur including stopcocks and no caps. Arrow marks CVC line

**Fig. 3:**
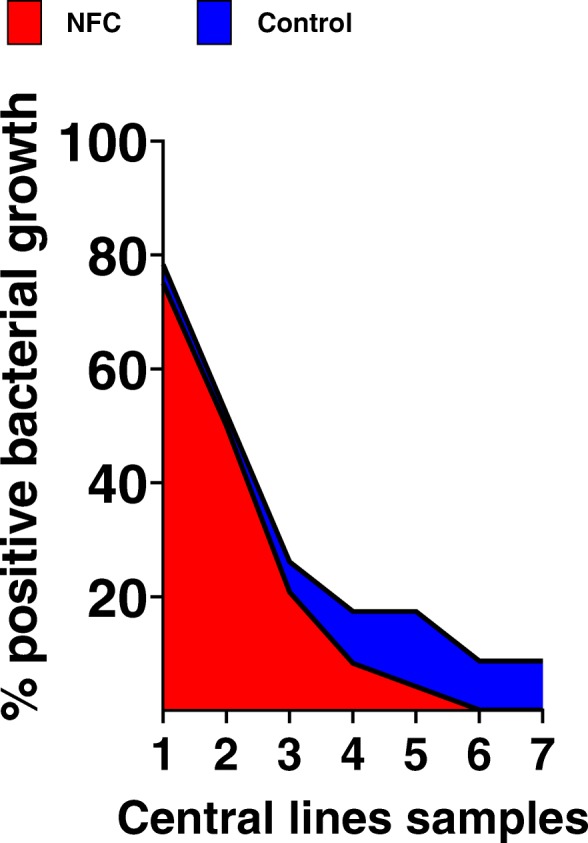
Bacterial contamination of entry ports. Percent of the bacterial growth of entry port sampling during sampling period

In our observational study, we next analyzed, if NFC changed the level of entry port contamination. For this, each entry port was sampled when infusion lines were changes. As shown in Table 3, coagulase-negative staphylococcus dominated the contamination of entry port of arterial lines. The distribution of microbiological genus was identical when the CVC contamination flora was analyzed: here, also the coagulase-negative staphylococci dominated the contamination of the entry ports (Table 4). Next, we analyzed the percentage of bacterial growth in microbiological samples taken from the entry ports of CVC. As shown in Fig. 2, 75% of CVC entry ports in the NFC group and 78.5% of the entry port in the control group were contaminated during the first programmed change of infusion lines. The infusion lines were renewed every 72 h and we acquired microbiological samples of each entry port on that occasion. Interestingly, the percentage of contaminated ports decreased over time, and no growth could be detected after 6 changes of infusion lines in the NFC group (Fig. 3).

**Table 3:** Microbiological results arterial catheter

	NFC (*n* = 96)	Controls (*n* = 92)	*p* values
No growth	130	113	0.16
Coagulase-negative staphylococci	19	16	0.85
Staphylococcus hominis	7	2	
Staphylococcus simulans	0	0	
Staphylococcus capitis	0	2	
Staphylococcus epidermidis	10	11	
Staphylococcus haemolyticus	2	1	
Streptococcus mitis	–	1	
Micrococcus luteus	1	–

**Table 4:** Microbiological results in central venous catheter

	NFC (*n* = 168)	Controls (*n* = 161)	*p* values
No growth	130	113	0.17
Coagulase-negative staphylococci	29	34	0.15
Staphylococcus hominis	4	8	
Staphylococcus simulans	1	0	
Staphylococcus capitis	2	2	
Staphylococcus epidermidis	14	18	
Staphylococcus haemolyticus	8	6	
Corynebacterium tuberculostearicum	1	0	
Enterococcus faecium	2	0	
Streptococcus mitis	1	2	
Streptococcus parasanguinis	1	1	
Streptococcus viridans spp	0	1	
Pantoea agglomerans	1	0	
Enterococcus faecalis	1	1	
Micrococcus luteus	1	2

According to our study protocol, every time an entry port sampling tested positive, a blood culture from a peripheral vein was collected. If a CRBSI was suspected based on clinical signs of infection, blood cultures were collected from a peripheral vein, the catheter was removed, and the catheter line tip underwent microbiological testing. In total, 47 central venous catheter line tips were cultured. We found identical pathogens in blood culture and CVC lines tips in two patients of the NFC group and one patient in the control group, making the diagnosis of CRBSI. Based on these numbers, the calculated device-associated infection rate was 1.83 and 3.8 per 1000 patient days in the control and NFC group, respectively.

Taken together, NFC use had no impact on bacterial contamination of entry port, but data indicate that the use of NFC decreases the rate of contamination over time faster when compared to controls.

## Discussion

In this randomized prospective observational trial, we found that NFC has no impact on bacterial contamination of intravascular catheter entry port or CRBSI rate.

Patients in surgical ICUs require CVCs for drug administration and hemodynamic monitoring, which put them at risk for CRBSI. The reported incidence rate of CRBSI by German National Reference Center for the Surveillance of Nosocomial Infections is 1.02 per 1000 catheter days in surgical ICUs [9]; reports from our ICU list an even lower incidence of (0.38/1000 catheter days; unpublished internal reports of our institution). However, these numbers warrant caution, since they often rely on the analysis of billing codes. By nature, data from reporting systems relying on billing codes can differ from data gathered in clinical trials. If CRSBI is underdiagnosed and undercoded, it will be underreported. It is therefore not surprising that in our clinical study, we found a much higher incidence rate of CRBSI (2.86/1000 catheter days) than expected. 

A risk factor for CVC-associated CRBSI is contamination of entry ports; thus,s interventions reducing contaminations could subsequently reduce rates of CRBSI. Holroyd showed that none of the routine procedures is efficient in decontamination of the inner conus of a stopcock, whereas the surface of the NFC could be decontaminated efficiently [10]. Furthermore, some of the agents used for disinfection like chlorhexidine [11, 12]) may include the risk for antibiotic resistance [11], NFC may represent a device with a lower risk for further resistance. In our study, disinfection was performed according to a recommendation by the manufacturer, nevertheless, we could find contamination at the rim of the CVC connector in 75% out of NFC swabs. Only in one patient of the NFC patients, pathogen present in the connector also leads to a blood-culture positive infection as compared to two CRBSI in the control group. This is in line with previous studies, where NCF also had no impact on the rate of bloodstream infections [13]. Since the healthcare-associated infections increase with the number of manipulations [14], this could explain our findings, since our study protocol reduced the number of programmed infusion line changes. This alone could have contributed to the relatively low numbers of CIRBS in our study.

NFC use in our study also did not increase the rate of contamination or CRBSI. This is in contrast to other findings results from retrospective case series [15–17], which found a spike in CRBSI after the introduction of NFCs and the United States Food and Drug Administration recommended to avoid NFCs. In part, this can be explained by the physical construction of the connector we used (Bionecteur®), which differs from previous models since it only allowed a retrograde flow of blood into connectors. This limits the chance of accidental injection of contaminated material present in the connector into the bloodstream. In vitro studies indicate that design only minimally impacts bacterial contamination, whereas the frequent use and usage time primarily determine if bacterial form colony within the connector of the NFCs [18]. In our study, we changed the NFC every 3 days together with the central and arterial lines, even though the manufacturer recommends the use for 7 days or 360 connections. This could also contribute to the low numbers of contaminations in the NFC group since in vitro studies suggest that bacterial load increases after 4 days regardless of the numbers of disinfections [18]. The use of stopcocks also bears the risk of bacterial contamination in the dead space [19], but rates were similarly low in the control group than in the NFC group.

In total, data from only 47 patients underwent analysis and thus the study cohort is not suitable to draw conclusions on the impact of rare events such as catheter-related bloodstream infections. This could be interpreted as a weak point of our trial. Yet, PubMed lists only two clinical trials investigating NFCs in the setting of adult critical care. Both trials were performed more than a decade ago [20, 21], when the physical construction of NFCs differed from the current models. Nevertheless, various authorities recommend the usage of NFCs to reduce intravascular catheter-associated infections despite uncertain benefits (e.g., German Commission on Hospital Hygiene, KRINKO in 2017). Thus, we feel that our trial provides information in a field with only scarce data to date—albeit based on a small study cohort.

## Conclusion

Taken together, NFC usage for intravascular infusion line port has no impact on bacterial contamination or CRSBI. However, there was a trend towards after sterile conditions in the NFC group, which warrant further investigation.

## Data Availability

All data included in this study are available from the corresponding author on reasonable request.

## Abbreviations

CVC: Central venous catheter CRBSI Catheter-related bloodstream infection ICU Intensive care unit KRINKO Kommission für Krankenhaushygiene und Infektionsprävention NFC Needle-free connector RKI Robert Koch Institute
